# Macro- and Microelement Composition, Antioxidant Activity, and Biological Effect of Cold-Pressed Edible Oils from Commercial and Amateur Companies

**DOI:** 10.3390/molecules30071425

**Published:** 2025-03-23

**Authors:** Jolanta Marciniuk, Beata Sadowska, Marzena Więckowska-Szakiel, Mateusz Borkowski, Jacek Zebrowski, Bronisław K. Głód, Kacper Marciniuk, Paweł Marciniuk

**Affiliations:** 1 Faculty of Sciences, Institute of Biological Sciences, University of Siedlce, Prusa 14, 08-110 Siedlce, Poland; jolanta.marciniuk@uws.edu.pl; 2 Department of Immunology and Infectious Biology, Faculty of Biology and Environmental Protection, University of Lodz, Banacha 12/16, 90-237 Lodz, Poland; beata.sadowska@biol.uni.lodz.pl (B.S.); marzena.wieckowska@biol.uni.lodz.pl (M.W.-S.); 3 Faculty of Sciences, Institute of Chemical Sciences, University of Siedlce, 3 Maja 54, 08-110 Siedlce, Poland; mateusz.borkowski@uph.edu.pl (M.B.); bkg@onet.eu (B.K.G.); 4 Institute of Biotechnology, College of Natural Sciences, University of Rzeszow, Pigonia 1, 35-310 Rzeszow, Poland; jaze28@interia.pl; 5 Faculty of Human Nutrition, Warsaw University of Life Sciences, Nowoursynowska 166, 02-787 Warsaw, Poland; kacper.marciniuk@o2.pl

**Keywords:** cold-pressed oils, macronutrients, micronutrients, total antioxidant potential, FTIR spectroscopy, antimicrobial activity

## Abstract

The aim of this study was to examine cold-pressed oils available on the Polish market derived from different plants and manufacturers in the context of their biological activity, including micro- and macroelements, antioxidant properties, antimicrobial activity, and selected effects on eukaryotic cells. In total, 76 oil samples of 34 selected oil types from nine Polish companies (five commercial and four amateur) were tested. The content of macro- and micronutrients was assessed using ICP-OES, the level of fatty acid unsaturation was examined using Fourier transform infrared spectroscopy (FTIR), and total antioxidant potential (TAP) was assessed using the DPPH method. The antimicrobial activity of the selected oils against Gram-positive and Gram-negative bacteria, as well as fungi, representing both pathogens and human microbiota, was tested using the broth microdilution method. The MTT reduction assay was used to exclude the cytotoxic effect of the oils on the human fibroblast line HFF-1. It has been concluded that the composition of cold-pressed oils varied significantly depending on the plant used and the manufacturer. The total content of the elements tested ranged from 172.91 mg/kg in *Helianthus annuus* oil to 1580.73 mg/kg in *Silybum marianum* oil. The iron concentration limits were exceeded in 10 oils, the copper concentration limits were exceeded in 34 oils, and the lead concentration limits were exceeded in 18 oils. At least one of these elements was exceeded in 40 oils (53% of the tested samples), which is why testing the concentration of elements should be a standard procedure for assessing the quality of cold-pressed oils. There was no statistically significant correlation between the content of any macro- and microelements and TAP. While TAP was strongly correlated with the spectral unsaturation index of the oils, this relationship can be used to develop a simple and rapid assessment of oils quality. The strongest antioxidant activity (over 90%) was observed for *Nigella sativa* oils. Interestingly, among all the tested oils, only these from *Nigella sativa* L., whatever the producer, possessed also strong antimicrobial activity. None of the tested oils showed cytotoxicity against eukaryotic cells, so the cold-pressed oils can be considered safe.

## 1. Introduction

Due to their content of unsaturated fatty acids, plant oils are an essential part of the human diet. In recent years, unrefined cold-pressed oils have gained significant popularity in the health food market. The global cold-pressed oils market is estimated to reach a value of USD 36.40 billion by 2026, compared to a value of USD 24.62 billion in 2018. This is attributed to the growing consumer interest in unprocessed food from natural sources, which is rich in antioxidant compounds. In European markets, alongside oils from traditional oilseeds like rapeseed, sunflower, flax, and soybeans, oils from seeds not commonly used on an industrial scale in Europe, such as tea, safflower, or poppy seeds, as well as from non-oil plants like carrots, grapes, nettles, roses, and walnuts, are becoming increasingly popular [1]. The cold-pressing procedure is technologically simple and ecological because it avoids heat and chemical treatment. The cold-pressed oils can only be purified by means of water, sedimentation, filtration, and centrifugation. The products obtained in this way have better nutritional properties and biological activity than the industrially produced refined oils [2]. The lack of high temperatures and chemical treatment during the entire technological process is responsible for the high content of biologically active compounds. Besides unsaturated fatty acids, phospholipids, tocopherols and tocotrienols, free and esterified sterols, hydrocarbons (squalenes), triterpene alcohols, carotenoids, and chlorophylls are described [3]. The chemical composition of the same grade oils from different manufacturers, and even from different batches of the same manufacturer, can differ significantly. It depends on many factors, such as geographical origin, cultivation methods, quality and storage conditions of seeds, as well as pressing parameters. Moreover, the oxidative stability of cold-pressed oils obtained from seeds is very often lower than their refined counterparts. This can be explained by the presence of pro-oxidant chemicals, e.g., metals (iron, copper, manganese), chlorophyll pigments, hydrolysis, and oxidation products. An additional factor that strongly affects the quality of oils is their improper storage; often, cold-pressed oils are poured into clear glass bottles and displayed in shops or at manufacturers in full light [4]. Cold-pressed oils are considered functional foods, not only because of the essential fatty acids they contain but also because of their antioxidant properties [3]. The rapidly growing demand for healthy food, which includes cold-pressed vegetable oils, has contributed to a significant increase in the range of products offered, lowering the quality or adulteration of these products. This especially applies to small producers, often operating outside of formal control.

Therefore, it becomes necessary to introduce low-cost, sensitive, simple, and fast methods to assess the quality of oils. Various spectroscopic methods, particularly infrared spectroscopy, have been established to match these demands.

Oil characteristics such as the iodine value, saponification number, peroxide value, and degree of oxidation appeared well predicted based on infrared spectra [5,6,7,8]. This technique itself or combined with chemometrics also enabled successful oil classification, the estimation of their origin, confirmation of authenticity, and monitoring adulteration [9,10,11,12].

This research was focused on assessing the composition and biological activity of the cold-pressed oils available on the Polish market. The goal was to determine the composition of cold-pressed oils derived from different plant sources and manufacturers in the context of their biological activity, including antioxidant properties, antimicrobial activity, and the effect on eukaryotic cells. Additionally, the potential of using FTIR spectroscopy for an indirect prediction of the total antioxidant potential (TAP) of cold-pressed oils was verified for the first time.

## 2. Results and Discussion

### 2.1. The Content of Macro- and Microelements in the Oils

The content of macro- and microelements of the oils tested by us (Table 1) is summarized in Tables S1 and S2 (Supplementary Materials). The total content of the examined elements ranged from 172.91 mg/kg in *Helianthus annuus* oil produced by a small amateur company to 1580.73 mg/kg in *Silybum marianum* oil also embossed by an amateur company. The content of macronutrients (Ca, K, Mg, P, Na, and S) in the tested oils was variable and probably depends, to a small extent, on the botanical origin of the oil. The highest total macronutrient content was found in *Silybum marianum* oil (average 644.87 mg/kg, with a wide range from 265.36 mg/kg to 1561 mg/kg) in one of the two *Sinapis alba* oils tested: 1242 mg/kg in *Cucurbita pepo* oil, average 555.03 mg/kg (in the range 319.83–1158.61 mg/kg), one of two *Cocos* oils 817.69 mg/kg, and *Cannabis sativa* oil, average 535.07 mg/kg (in the range of 268.83–938.55 mg/kg). The content of macronutrients over 400 mg/kg was also found in the following oils: *Linum usitatissimum* (range 210.04–587.69 mg/kg), *Brassica napus* (range 208.82–543.3 mg/kg), and *Nigella sativa* (range 244.87–665.9 mg/kg). The analysis of the average macronutrients content of in all of the tested oils showed that Na was the most abundant element—143.44 mg/kg (with the range from 92.91 mg/kg in *Hippophae rhamnoides* oil to 286.99 mg/kg in *Silybum marianum* oil from a commercial company). The Ca content was the second numerous—89.43 mg/kg (with a very large range from 5.96 mg/kg in organic *Glycine max* oil to 477.04 mg/kg in *Sinapis alba* oil from a commercial company), and then P—71.48 mg/kg (with very high variability from 12.94 mg/kg in *Hippophae rhamnoides* oil to 523.31 mg/kg in *Cucurbita pepo* oil from a commercial company). The remaining macronutrients were less abundant: K—50.59 mg/kg (in the range from 1.09 mg/kg in *Nigella sativa* oil from a commercial company to 308.7 mg/kg from a small amateur company), S—50.46 mg/kg (with a range from 0.58 mg/kg in *Helianthus annuus* oil from amateur company to 142.93 mg/kg in *Cocos* oil from a commercial manufacturer), and Mg—28.64 mg/kg (with a range from 3 mg/kg in *Helianthus annuus* oil from a commercial company to 155.27 mg/kg in *Cucurbita pepo* oil from a commercial company). The proportions of macronutrients content in individual oils often differed from the average values, e.g., in 14 oils, the highest content of calcium was found, in 5 with potassium and in 5 with phosphorus (Table S1). An interesting result was the clearly lower (below average) content of macronutrients in the oils marked as ecological, produced by the large commercial companies operating in the Podlachia region (north-eastern Poland). Most of the raw materials were obtained from ecological farms operating in this region, cooperating with the companies based on long-term contracts. The relatively low content of macroelements in ecological oils may result from the synergy between extensive agriculture and the naturally poor fertility of soils in this region. The average content of micronutrients (Zn, As, Fe, Cu, Se, Mn, Cd, Pb, Mo, Cr, Se) in the tested oils was as follows (Table S2): zinc was the most abundant—3.36 mg/kg (with a range of 0.69 mg/kg in the oil *Carthamus tinctorius* up to 8.79 mg/kg in *Cannabis sativa* oil) and then iron—3.31 mg/kg (with the range from 1.12 mg/kg in *Cocos oil* to 8.70 mg/kg in *Silybum marianum* oil). The manganese content in the analyzed oils was low (partially below the limit of quantification). The presence of this micronutrient was found in 14 oils—8 *Cannabis sativa* oils (range 1.11–2.18 mg/kg), 3 *Silybum marianum* oils (range 0.1–1.89 mg/kg), 1 *Camelina sativa* oil (1.08 mg/kg), 1 *Cocos* oil (3.03 mg/kg), and 1 *Sinapis alba* oil (7.12 mg/kg). The copper content in the tested oils did not exceed the value of 1 mg/kg. The exceptions were one *Silybum marianum* oil (1.08 mg/kg), one *Carthamus tinctorius* oil (1.14 mg/kg), *Prunus armeniaca* seeds oil (1.05 mg/kg), and *Trigonella foenum-graecum* oil (1.28 mg/kg). Pb was detected in twenty oils (concentration range from 0.04 to 0.931 mg/kg), i.e., five Cannabis sativa oils, three *Silybum marianum* oils, four *Brassica napus* oils, three *Helianthus annuus* oils, one *Cucurbita pepo* oil, one *Borago officinalis* oil, *Prunus armeriaca* oil, *Daucus carota* ECO oil, and *Urtica dioica* ECO oil. The remaining micronutrients in the tested oils were found in trace amounts. Macro- and micronutrients play an important role in many physiological functions of the human body [13]. Cold-pressed oils are considered functional food due to the presence of unsaturated fatty acid (MUFA and PUFA) polyphenols, tocopherols, and other biologically active compounds [14]. The content of macro- and microelements in oils was examined much less frequently. In the oils produced in China tested for the content of heavy metals [15], a lower content of zinc, copper, and manganese and a significantly higher content of iron were found compared to these findings. The use of defective or worn presses might be a reason for such increased iron content, which can be explained by the use of defective or worn oil presses. Several authors indicated the possibility of the contamination of oils with metals, especially iron, during the pressing process [16]. In this study, the ranges of micronutrient and macronutrient content in the oils of the same type were broad and did not differ significantly from already published data [17]. In cold-pressed oils, the maximum concentration limits for Fe, Cu, Pb, and As have been established, which are 5 mg/kg, 0.4 mg/kg, 0.1 mg/kg, and 0.1 mg/kg, respectively [18,19]. In the oils examined in this study, the concentration limit of at least one of the three elements (Fe, Cu, and Pb) was exceeded in 40 oils (53% of the tested samples) pressed by both commercial and amateur producers (Table S2). The concentration limit for iron was exceeded in 10 oils, the concentration of Cu was exceeded in 34 oils, and the concentration of lead was exceeded in 18 oils. Interestingly, the concentration limit was exceeded in four oils (two *Cucurbita pepo* and two *Silybium marianum*) pressed by amateur companies. The reason is probably the producers’ desire to reduce costs by buying the cheapest raw material from intensively fertilized and sprayed crops. The results obtained indicate that testing the concentration of elements should be a standard procedure for assessing the quality of cold-pressed oils.

### 2.2. Total Antioxidant Potential (TAP)

**Figure 1:**
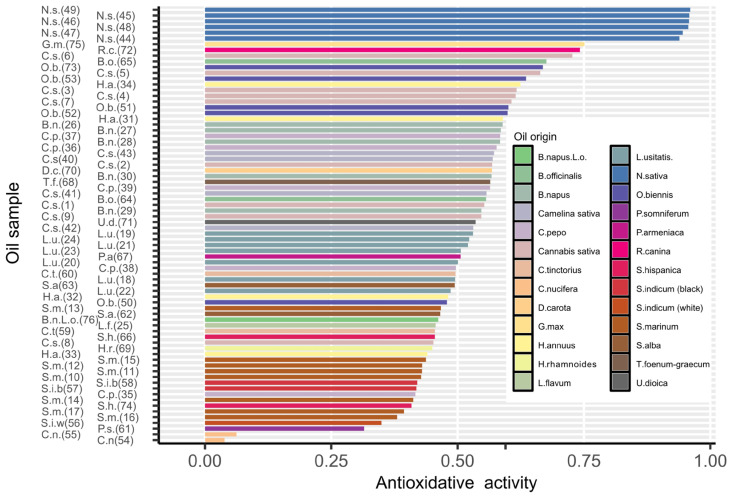
The total antioxidative activity for oil samples in increasing order of values. Numbers in brackets placed after the initials of the species indicate the sample number (Table 1). The values presented were determined with a standard deviation not exceeding 3%.

The total antioxidant potential of edible oils was examined using the DPPH assay. This is one of the most commonly used methods for determining the antioxidant properties of food because it is simple, quick, and highly reproducible. It involves using a stable free radical, DPPH (2,2-diphenyl-1-picrylhydrazyl), which reacts with antioxidants present in food samples. The method is also advantageous because it can be applied to a wide variety of food matrices, does not require complex equipment, and provides precise, measurable results through a change in color that can be easily monitored spectrophotometrically. Additionally, it is cost-effective and suitable for screening large numbers of samples in a relatively short amount of time [20]. Similar methods, using other radicals (oxidants) such as AAPH, MPTP, ABTS, or NO, give comparable results due to their similar standard redox potentials. To obtain additional information, more potent oxidants, e.g., hydroxyl radicals, should be used [21]. Unfortunately, such assays can be used only in aquatic environments, because this radical oxidizes alcohols and other organic solvents. For the DPPH assay, the most common solvent is methanol, but there is a possibility of using n-propanol, carbon tetrachloride, 1,4-dioxane, or methyl acetate depending on the matrix [22]. Changing methanol for propanol does not negatively influence the DPPH assay, as proved in other studies [23,24]. In this study, methanol was changed for n-propanol because coconut oils did not fully dissolve in methanol. The total antioxidant potential of most of the tested oils exceeded 50% (Table S3, Figure 1). Exceptionally high TAP was found in *Nigella sativa* oils—an average of 95.29% (with a range of 93.91–96.04%). A TAP value exceeding 70% was found in ecological oils: *Glycine max* mineral oil (75.10%) and *Rosa canina* oil (74.22%), as well as in one of the *Cannabis sativa* oil (72.71%). The average TAP value of *Cannabis sativa* oils was 59.44% with a relatively large range from 45.21 to 72.71%. Relatively high values of TAP were found in several oils. In *Oenothera biennis*, the average value was 59.65%, ranging from 47.87% to 66.83%. The oil from *Borago officinalis* had values of 67.55% and 55.63%. For *Brassica napus*, the average was 57.45%, ranging from 54.72% to 58.90%. In *Camelina sativa*, the average was 55.75%, ranging from 53.11% to 57.22%. The oil of *Helianthus annuus* showed an average of 53.41%, with an extensive range from 44.04% to 62.43%. The average for *Cucurbita pepo* was 52.77%, with a range of 41.67% to 58.41%. For *Linum usitatissimum*, the average was 50.89%, with a range of 48.63% to 53.06%. Additionally, relatively high values were found in *Daucus carota* oils at 56.81%, *Trigonella foenum-graecum* at 56.44%, *Urtica dioica* at 53.56%, and *Prunus armeniaca* at 50.61%. In the case of other oils, TAP did not exceed 50%. *Cocos* oils have approx. zero antioxidant potential. Zemour et al. [25] published data similar to the results obtained in this study. Cited work confirmed the very high antioxidant activity of *Nigella sativa*, *Glycine max*, *Cannabis sativa*, *Rosa canina*, and *Brassica napus* oils. The extremely low activity of *Cocos* oil, sold by Polish commercial companies as cold-pressed oil, is quite surprising. Comparative studies on the antioxidant activity of cold-pressed and other refined, bleached, and deodorized coconut oils have shown that untreated cold-pressed coconut oils have quite a high antioxidant activity [26,27]. It is possible that the coconut oils we tested were pressed from low-quality raw material and might have undergone additional processing.

### 2.3. Correlations Between the Content of Macro- and Micronutrients and Total Antioxidant Potential (TAP)

**Figure 2:**
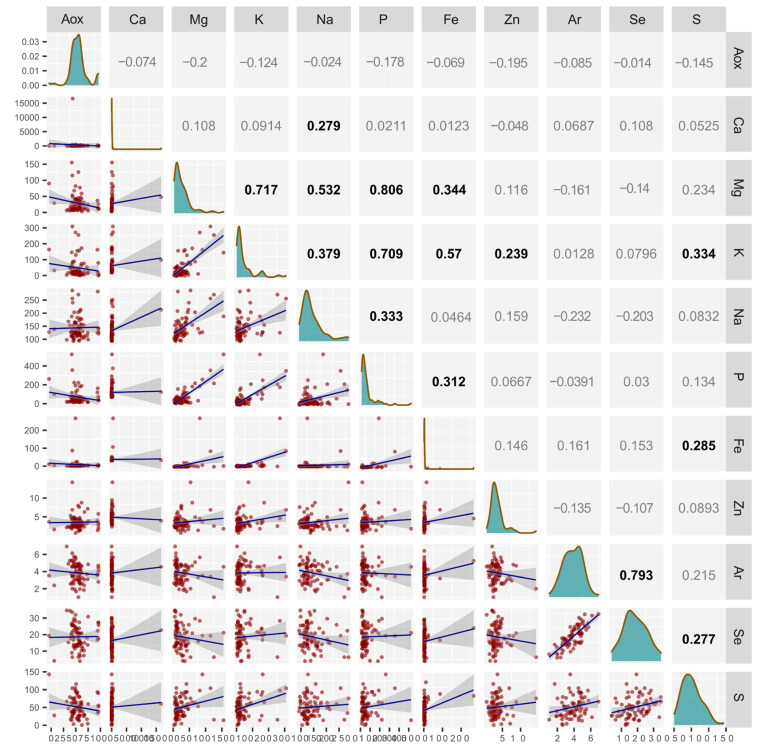
The correlation matrix showing the relationship between the content of macro- and micronutrients and the total antioxidant potential (Aox). Significant (alpha = 0.05) Pearson correlation coefficients are marked in bold.

The relationship between micro- and macroelements, as well as between TAP and the presence of micro- and macronutrients in the tested oils, is shown in Figure 2.

The analysis omitted copper and manganese, which were below the quantification level in some oils. Statistically significant positive correlations were found between the content of calcium and Mg, K, P, Na, Zn, Fe, and S. Phosphorus was positively correlated with Ca, Mg, K, Na, and Fe, while sodium was positively correlated with Ca, Mg, K, and P. Iron was positively correlated with all macronutrients, the sulfur content in oils was positively correlated with Ca, K, Fe, and Se, zinc was positively correlated with Ca and K, and selenium was positively correlated with sulfur and arsenic. No statistically significant correlation existed between the content of any elements and TAP. However, a statistically significant relationship between the content of elements, especially zinc, and the antioxidant properties of honey was found by Bodo et al. [28]. Zinc and, to a greater extent, selenium are microelements with confirmed antioxidant properties [29,30]. In the case of fats, selenium plays a particularly important role due to its synergistic effect with vitamin E being one of vegetable oils’ most important antioxidants [27]. Selenium is a component of the prosthetic group of glutathione peroxidase (GPx1, GPx2, GPx3, and GPx4) [31,32]. Therefore, their antioxidant action is indirect. Both metals do not react with DPPH. The lack of correlation between the content of selenium and TAP in this study probably results from slight differences and a low content of this element in the analyzed oils and the activity of other antioxidants. A high concentration of trace elements in edible oils may lower their quality due to heavy metals or arsenic toxicity. However, no exceedance of toxicity thresholds for any of the potentially harmful elements for humans has been reported in edible oils [33]. A more serious threat to the quality of oils is the pro-oxidative activity of microelements, especially copper and iron, which may adversely affect the oxidative stability of oils and shorten their shelf life [34]. Their pro-oxidative properties are related to the catalytic effect of Fe^II^ and Cu^I^ with hydrogen peroxide (Fenton and Haber-Weiss reactions) [35,36]. Positive correlations between the content of elements, especially macronutrients, in the analyzed oils indicate the companies’ use of plant material from multicomponent fertilized crops. The close correlation between selenium and arsenic is interesting. Arsenic, even at low concentrations in food, is a highly toxic element that can cause serious diseases [37], but some positive effects are reported. For example, low arsenic levels can positively affect the heart (alter QT interval duration) [38]. On the other hand, selenium is a very important micronutrient for human and animal health, but it has a low toxicity threshold [39]. Selenium intake recommended by the European Food Safety Authority (EFSA 2014) [40] for adults is 70 μg Se per day. In Poland, the consumption of this element is clearly below the norm (30–40 μg Se per day) [41]. With increasing environmental pollution, selenium deficiencies may also be a serious problem because of the proven properties of selenium in neutralizing the toxicity of heavy metals and arsenic [42].

### 2.4. Spectral Index of the Degree of Oil Unsaturation

**Figure 3:**
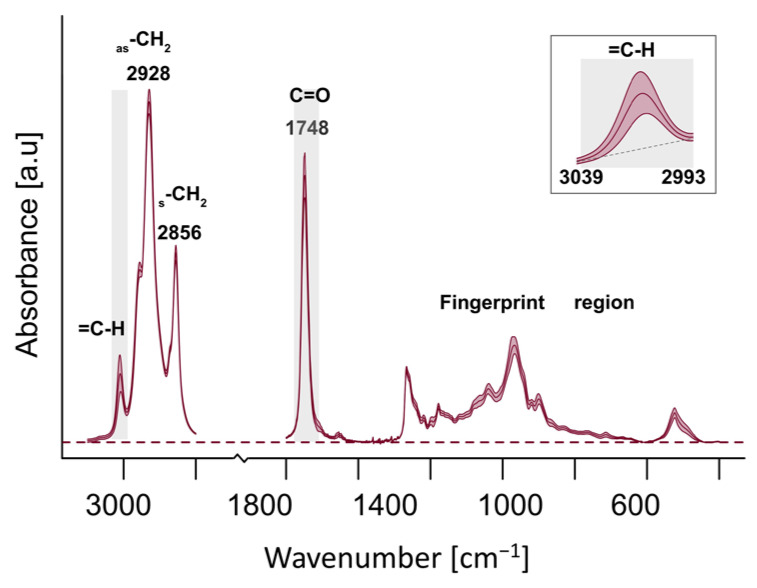
ATR-FTIR spectra of analyzed set of oils (mean (inner line) and SD (ribbon)). Major bands are labeled with the wavenumber value and the mode of vibrations. Bands relevant for the calculation of the degree of fatty acid unsaturation are gray-shaded. The inset shows variability in the absorbance assigned to (=C-H) mode of vibrations (mean and SD) under magnification.

Mean values and SD of ATR-FTIR spectra, which were the basis of the calculation of the degree of fatty acid unsaturation in oils, are presented in Figure 3. The oils showed typical infrared spectral features [6,8,43,44] and were characterized by major bands at 2928 cm^−1^ and at 1748 cm^−1^, attributed to the asymmetric C-H stretching vibrations of methylene groups and the ester carbonyl group (C=O) of the triglycerides, respectively. The band around 3008 cm^−1^, which corresponded to the C-H stretching at the cis-double carbon (C=C) linkage, reflected the unsaturation degree of fatty acid chains [43,44].

**Figure 4:**
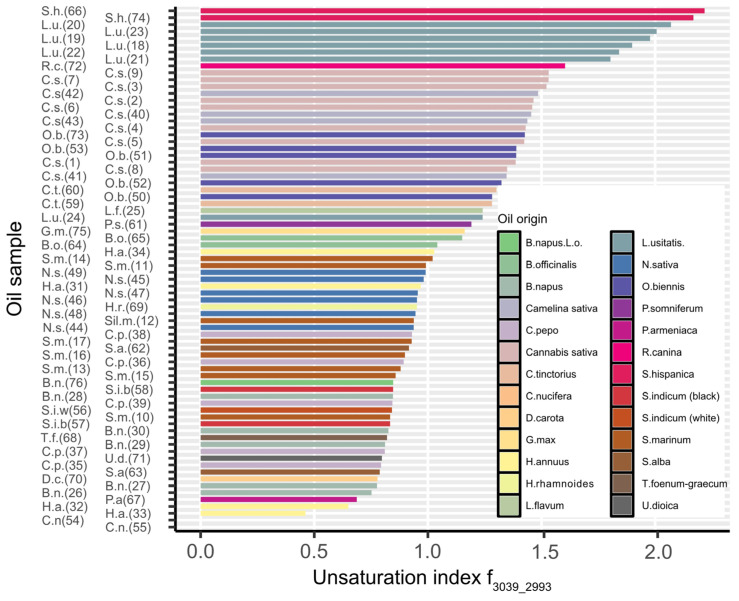
The index of the degree of fatty acid unsaturation calculated as summarized absorbance of the band between 3039 and 2993 cm^−1^ assigned to the =C-H stretching vibration. Numbers in brackets placed after the initials of the species indicate the sample number (Table 1). The values presented were determined with a standard deviation not exceeding 3%.

The rank of oils in terms of the spectral index, based on the I_3039_-I_2993_/I_1760_-I_1710_ absorbance ratio, is presented in Figure 4. *Salvia hispanica* oil (no. 66, 74) and *Linum usitatissimum* oil (no. 18, 19, 20, 21, 22, 23) were characterized by the highest values of the parameter. Oils of *Canabis sativa*, *Rosa canina*, and *Oenothera biennis* showed only a slightly lower index; *Cocos* oils were definitely at the bottom of this rank. In the case of some species, clear differences in the index were found between the oils, depending on their origin, that particularly concerns *Helianthus annus*, *Nigella sativa*, and *Brassica napus*.

**Figure 5:**
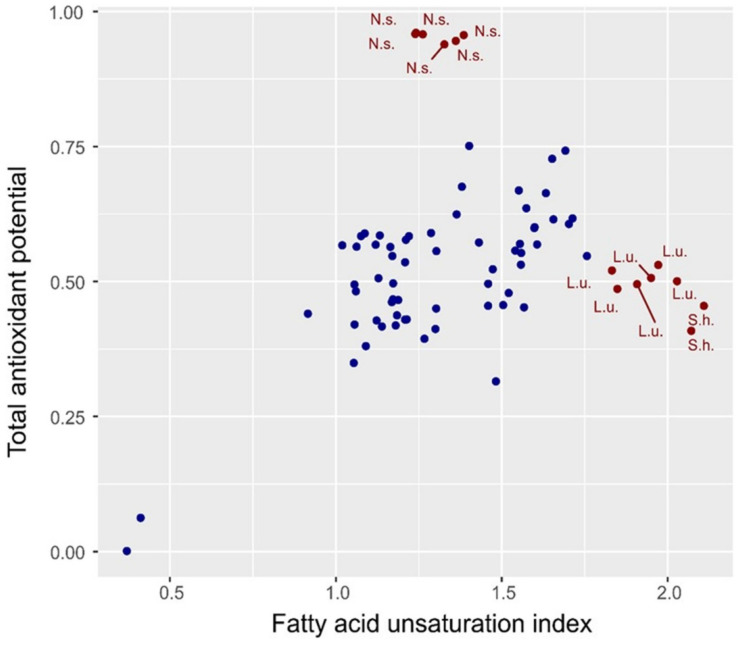
Scatterplot visualizing the relationship between the fatty acid unsaturation index, determined on the base of infrared spectroscopy, and the total antioxidant potential of oils. Red points and corresponding labels indicate species that deviate from the linear relationship between both parameters. High correlation coefficient (Pearson’s method) characterizes remaining (i.e., 90%) species (blue color), r = 0.684, CI = (0.524, 0.797), *p* < 0.001. N.s—*Nigella sativa* L., L.u.—*Linum usitatissimum* L., and S.h.—*Salvia hispanica* L.

Figure 5 shows a relationship between the spectral index of oil unsaturation and the total antioxidant potential (TAP). Generally, both parameters showed a linear relationship for most (90%) species. *Nigella sativa* was overestimated, while two species with the highest values of TAP were underestimated by the spectral index. This indicates that the degree of FA unsaturation determines to a large extent the total antioxidant potential. However, in the case of some species, other features may modify the TAP. These observations indicate the potential of infrared spectroscopy (IR) as a fast, simple, and non-destructive method that does not require special sample preparation for the indirect assessment of oil quality with respect to TAP. Developing a more effective model for TAP prediction would require extending spectra regions and combining these features with chemometrics, as it has been similarly shown for other oil characteristics like the iodine value, the saponification number, the peroxide value, and the degree of oxidation [5,6,7,8].

### 2.5. Antimicrobial Activity of the Oils

Antimicrobial activity was tested to assess the minimum inhibitory concentration (MIC) and minimum bactericidal/fungicidal concentration (MBC/MFC) of selected oils (n = 16, Table 2). At the same time, the MIC and MBC/MFC of the chosen antibiotics, oxacillin (Oxa), gentamycin (Gen), and fluconazole (Flu), against tested bacterial and yeast strains were also studied as a control of their susceptibility. The obtained results are presented in Table 2.

To verify the accuracy of the microbial response to biostatic/biocidal agents, the MIC and MBC/MFC of chosen antibiotics, including oxacillin (Oxa) against staphylococci, gentamycin (Gen) against *E. faecalis* and *E. coli*, and fluconazole (Flu) against *C. albicans*, were tested. All microbial strains responded with the following MIC/MBC or MFC values [µg/mL]: *S. aureus* 0.125/1 (Oxa), *S. epidermidis* 0.25/0.5 (Oxa), *E. faecalis* 8/32 (Gen), *E. coli* 1/2 (Gen), *C. albicans* >16/>16 (Flu). Among tested oils, only those obtained from *Nigella sativa* L. possessed strong biostatic and biocidal activity, mainly against Gram-positive bacteria, including *S. aureus*, *S. epidermidis*, and *E. faecalis* (Table 2). The MIC and MBC concentrations differed slightly depending on the microbial strain (better activity against staphylococci) and extract producer (company). However, all *N. sativa* oils (no. 44, 46, 48, and 49) had such antimicrobial activity. These oils also inhibited the growth of *C. albicans* with MIC in the range of 0.78–6.25% depending on the oil producer (Table 2). Yeasts were even effectively killed by *N. sativa* oils (oil no. 48 with MFC = 3.12% possessed the best fungicidal activity). Meanwhile, the rest of the oils did not exhibit biostatic and biocidal activity at the whole concentration range tested (up to 25%). Interestingly, *N. sativa* oils did not stand out in terms of macro- and micronutrient content compared to others (Tables S1 and S2), but they were characterized by the highest antioxidant potential of all the oils tested (Figure 1). The secondary metabolites of plants present in plant extracts, such as phenolic acids, flavonoids, catechins, tannins, cumarins, or carotenoids, as well as plant essential oils, possess a broad range of biological activities exerting pro-health effects. Between them, both antioxidative and antimicrobial activities are most often and together described [45,46,47,48,49,50,51]. Except classic antioxidants present in plant material, such as ascorbic acid or vitamin E, both the antioxidative and antimicrobial properties of plant-origin products are usually attributed to polyphenols [45,47,52,53,54]. Since polyphenols are also present in cold-pressed oils [14], they are probably responsible for the antioxidative and antimicrobial activities of these products. Based on our results for *Nigella sativa* oils (the best biological activity without distinct differences in macro- and micronutrients compared to other tested oils), we can speculate that these products may contain a high concentration of polyphenols. However, it requires further extensive research into the biochemical composition of tested oils.

### 2.6. Cytotoxic Effect of the Oils

**Figure 6:**
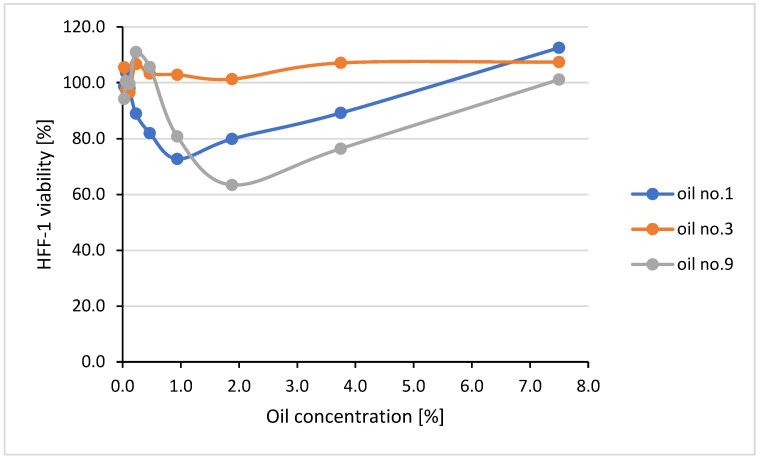
Cytotoxic activity of *Cannabis sativa* L. oils to human foreskin fibroblasts line HFF-1 tested by MTT reduction method.

The cytotoxic effect of the oils based on the activity of *Cannabis sativa* L. oils (no. 1, 3, and 9) against human foreskin fibroblasts line HFF-1 was tested using the MTT reduction method. The obtained results are presented in Figure 6 as the percentage of alive, metabolically active fibroblasts after 24 h of exposure to the tested oils used at different concentrations compared to the viability of untreated control cells considered at 100%.

It was demonstrated that oil no. 3 did not at all limit the fibroblast viability in the whole range of concentrations tested (up to 7.5%). Also, oils no. 1 and 9 were not cytotoxic (the HFF-1 viability did not fall below 80%) at most of the tested concentrations. Unexpectedly, the viability of HFF-1 dropped a little below 80% when the cells were exposed to oil no. 1 at a concentration of 0.94% and to oil no. 9 at concentrations of 1.88% and 3.75%. However, since the cell viability returned to 100% after the treatment with oils no. 1 and no. 9 used at higher concentrations, all of the tested oils can be considered safe (non-cytotoxic) for eukaryotic cells. At higher concentrations (12.5–50%), the oils formed a layer on the surface of the cell culture medium, which physically limited oxygen availability and led to cell death (the cell viability was 78.8–13.7%, 43.6–41.7%, and 54.7–33.8% for oils no. 1, 3, and 9, respectively, used at the concentration range of 12.5–50%).

## 3. Materials and Methods

### 3.1. Tested Oils

Cold-pressed vegetable oils were obtained directly from producers operating in various regions of Poland. Two categories of companies were deliberately selected, i.e., large commercial companies whose oils are available in retail chains throughout the country and small, semi-professional companies offering their products for direct sale (only from the manufacturer). We purchased all the types of cold-pressed oils produced by each company (Table 1). The oils for testing were stored in refrigerated conditions, protected from light. This publication is not intended to advertise any of the companies; therefore, their names and addresses will not be disclosed.

### 3.2. Total Antioxidant Potential (TAP)

Instrumentation

Total antioxidant potential (TAP) measurements were performed with the use of the Epoch Microplate Spectrophotometer (BioTek Instruments Inc., Winooski, VT, USA) equipped with a 96-well Agilent microplate and controlled with the Gen5 2.0 Software interface.

Reagents

2,2-diphenylo-1-picrylhydrazyl (DPPH) was obtained from Sigma (St. Louis, MO, USA), and 1-propanol was obtained from CHEMPUR (Piekary Śląskie, Poland).

Procedures

Edible oil samples (3 mg/mL) and DPPH were dissolved in 1-propanol. The DPPH free radical scavenging activity of the oil extracts was determined using a photometric method. For each measurement, 30 µL of the propanolic oil solution, 70 µL of 1-propanol, and 200 µL of a 1 mM DPPH solution were added to the wells of the microplate. The reaction mixture was thoroughly vortexed, and absorbance was recorded at 517 nm using the spectrophotometer. To account for background interference, a reference sample containing only 1-propanol was measured, with the oil sample replaced by 1-propanol. The decrease in absorbance was monitored at 20 min intervals until a stable reading was obtained, indicating that the reaction had reached equilibrium [55].

The total antioxidant potential (TAP) was calculated as the percentage decrease in absorbance using the following equation:

$${TAP}^{DPPH}=\frac{A_{DPPH}-A_{i}}{A_{DPPH}-A_{0}}\cdot 100\%$$

where *A_DPPH_* means the absorbance of the DPPH solution, *A_i_* is the absorbance of the oil solution after the reaction with the DPPH radical, and *A*_0_ is a blank absorbance.

Data Analysis

The measurements of the antioxidant capacity were performed three times for each sample, and the average was used for further calculations. Against the background of such obtained results (without sample), TAP values were subtracted.

### 3.3. Macro- and Microelements’ Analysis

The ICP-OES Perkin Elmer Optima 8300 spectrometer was used for the analysis, and the proprietary method of the MULTI 20 spectrophotometer was used. After the samples and the standard curves were prepared, they were manually entered into the spectrophotometer, and the elemental composition was calculated from the previously prepared standard curves. The coefficient of determination (R^2^) for the standard curves was 0.999. The spectrometer was operated using the Syngistix program.

### 3.4. ATR-FTIR Spectroscopy

Fourier transform infrared (FTIR) spectroscopy of oil samples was performed in the Attenuated Total Reflectance (ATR) mode using the iZ10 module of the Nicolet iN10 MX infrared imaging microscope (Thermo Fisher Scientific, Waltham, MA, USA), equipped with a deuterated triglycine sulfate (DTGS) detector and a KBr beam splitter.

A drop of oil sample was applied to the surface of the one-bounce diamond ATR crystal, previously carefully cleaned with isopropanol. Sixty-four spectra were collected at the 4 cm^−1^ resolution within the wavenumber range between 600 and 4000 cm^−1^ and averaged using OMNIC software (v.9.0, Thermo Fischer Scientific Inc. Waltham, MA, USA). Baseline corrected spectra were then normalized to unit area (1770–1690 cm^−1^; carbonyl C=O group region) using the ChemoSpec [56] package in the R programming language [57]. 

The ratio of absorbance between 3039 and 2993 cm^−1^ relative to that between 1760 and 1710 cm^−1^ (I_3039_-I_2993_/I_1760_-I_1710_), corresponding to the (=C-H) stretching and the (C=O) stretching vibrations, respectively, was considered to be the unsaturation index of fatty acids for the examined oils.

### 3.5. Microorganisms and Culture Conditions for Antimicrobial Activity Testing

The reference strains of bacteria and fungi representing physiological microbiota and pathogens of skin and mucous membranes: *Staphylococcus aureus* ATCC 29213 (MSSA, methicillin-susceptible *S. aureus*), *Staphylococcus epidermidis* ATCC 12228, *Enterococcus faecalis* ATCC 29212, *Escherichia coli* ATCC 25922, and *Candida albicans* ATCC 10231 were used to evaluate the biostatic/biocidal activity of selected oils. Bacteria were grown on tryptic soy agar (TSA; BTL Sp. z o.o. Lodz, Poland), while yeasts were grown on Sabouraud dextrose agar (SDA; BTL, Lodz, Poland) for 24 h at 37 °C. Then, microbial suspensions (2–8 × 10^5^ CFU/mL) were prepared in Mueller–Hinton broth (MHB; BTL, Lodz, Poland) or in RPMI-1640 medium with L-glutamine (Sigma-Aldrich/Merck, Darmstadt, Germany) containing 2% glucose (RPMI/Glu) for bacteria or fungi, respectively.

### 3.6. Assessment of Minimum Inhibitory and Bactericidal/Fungicidal Concentration (MIC, MBC/MFC)

The broth microdilution method was used to determine the MIC of the oils according to the EUCAST guidelines [58]. The solutions of the tested oils at the final concentration range (25–0.39%) (for selected oils and bacterial strains, the lower concentrations up to 0.01% were also tested) were prepared in MHB with 1% Tween 80 (Sigma-Aldrich/Merck, Germany) for bacteria or in RPMI/Glu with 2% Tween 80 for fungi. To 96-well culture plates (Falcon, Dublin, OH, USA), 100 µL of each oil solution and 100 µL of microbial suspensions (2–8 × 10^5^ CFU/mL) were added, and the plates were incubated at 37 °C for 24 h. Appropriate growth controls of the microorganisms were simultaneously prepared: K1—microbial suspensions (2–8 × 10^5^ CFU/mL) in culture medium alone (MHB or RPMI/Glu); K2—microbial suspensions (2–8 × 10^5^ CFU/mL) in culture medium (MHB or RPMI/Glu) containing finally 0.5% Tween 80 (bacteria) or 1% Tween 80 (fungi). Negative controls were culture media alone. The effect of the selected antibiotics on the tested microorganisms was also assessed: oxacillin (Oxa; 2-0.015 µg/mL) against staphylococci, gentamycin (Gen; 32-0.25 µg/mL) against *E. faecalis* and *E. coli*, and fluconazole (Flu; 16-0.25 µg/mL) against *C. albicans*. MIC was defined as the lowest concentration of tested oils inhibiting visible bacterial/fungal growth during co-incubation time compared to the appropriate growth control. MBC/MFC of the oils referred to the lowest concentration that killed 99.9% of microbial inoculum added to the wells. Thus, no bacteria or yeast grew after subculturing 10 µL from the wells tested on TSA/SDA (incubation 24–48 h at 37 °C). Experiments were carried out at least in duplicate for each oil and each microbial strain.

### 3.7. Evaluation of Cytotoxicity for Human Fibroblasts

The cytotoxicity of the selected oils was tested for human foreskin fibroblasts line HFF-1 (ATCC-SCRC-104, LGC Standards, Kiełpin, Poland) using the MTT [3-(4,5-dimethylthiazole-2-yl)-2,5-diphenyltetrazolium bromide] (Sigma, St. Louis, MO, USA) reduction method, as recommended by the ISO norm 10993-5 [59]. The cells were cultured in whole culture medium (WCM) containing Dulbecco’s Modified Eagle’s Medium high glucose (DMEM hg), 15% fetal calf serum (FBS), and penicillin/streptomycin (P/S, 100 U/mL/100 μg/mL, 100× concentrate), all from BioWest, France. Cell suspension (5 × 10^5^ cells/mL) in WCM was seeded (100 μL) into 96-well tissue culture plates (Nunc, Roskilde, Denmark) for 24 h at 37 °C/5%CO_2_ to form a confluent cell monolayer. Initial solutions of the oils (75% in DMSO) were diluted 10 times in WCM, and next, a series of 2-fold dilutions was made (the oils concentration range tested: 7.5-0.029%). In this study, the oils in a higher concentration range (50–12.5%) were also prepared in WCM starting from 100% oils. The cell monolayers were washed with phosphate-buffered saline (PBS; BioWest, France) and exposed to WCM containing the tested oils at concentration ranges of 7.5–0.029% and 50–12.5% (100 μL/well) for 24 h at 37 °C/5%CO_2_. Appropriate cell growth controls were set up at the same time: K1—the cells in WCM alone, K2—the cells in WCM with 1.25% DMSO, and K3—the cells in WCM with 2.5% DMSO, which was the highest possible concentration of DMSO in the tested samples. After exposure, the medium containing oils was removed, the cells were washed with PBS, and then fresh WCM (100 µL/well) and MTT (1.5 mg/mL, 50 µL/well) were added for 2 h of incubation under the same conditions. Finally, MTT solution was removed and replaced with 100 μL/well of 20% sodium dodecyl sulfate (SDS; Sigma, St. Louis, MO, USA) for 24 h (room temperature) to dissolve the blue formazan crystals formed in the presence of living cells. The absorbance of the samples was read at λ = 550 nm using a microplate reader (Victor2, Wallac, Turku, Finland), and the percentage of viable cells was calculated compared to the appropriate growth control considered at 100% cell viability. The experiment was prepared twice in technical duplicates each.

### 3.8. Statistical Analysis

Infrared spectroscopy data and derived characteristics were depicted by means of R programming language (R Core Team, 2023) [57] using the ChemoSpec [56] and ggpubr [60] packages.

Similarity between the spectra, with respect to the unsaturation indices, was determined using the distance function and Pearson’s method. The “distance” function was also used to show similarities between the oils for the total antioxidative potential.

The correlation matrix with significance levels was obtained on the basis of the Hmisc package [61].

## 4. Conclusions

A wide range of cold-pressed oil types was studied to evaluate the impact of biological (28 cultivars) and manufacturer (9 companies) origin on selected biochemical and biological traits determining their quality. The set of examined oils covered almost the total assortment of edible oils available on European markets. The measured traits involved the total antioxidant potential, the content of macro- and microelements, the degree of fatty acid unsaturation, and antimicrobial activity against Gram-positive and Gram-negative bacteria and against fungi representing both pathogens and human microbiota. Additionally, the cytotoxic effect against human fibroblasts line HFF-1 was also assessed for the selected oils.

This study showed that the content of macro- and microelements in the oils depended on their botanical origin. Sodium, potassium, and phosphorus among macronutrients, and iron and zinc among the trace elements, showed the highest content.

The iron concentration limits were exceeded in 10 oils, the copper concentration limits were exceeded in 34 oils, and the lead concentration limits were exceeded in 18 oils. At least one of these elements was exceeded in 40 oils (53% of the tested samples), which is why testing the concentration of elements should be a standard procedure for assessing the quality of cold-pressed oils.

Very high and high total antioxidant potential (TAP) (above 50%) was demonstrated in 15 types of oils. The exceptionally low value of oil unsaturation showed *Cocos* oil, while the remaining differed by more than four-fold, depending on origin.

The use of infrared spectroscopy showed a linear relationship between the spectral index of oil unsaturation and TAP for most of the oil types, which indicates the potential of this fast, simple, and low-cost method for throughput-routine evaluation of the parameter. Potentially, this method can be used for a rapid evaluation of the quality of cold-pressed oils. However, further studies are needed to standardize the method.

Interestingly, among all of the tested oils, only those from *Nigella sativa* L., whatever the producer, also possessed strong antimicrobial activity. None of the tested oils showed adverse effects (cytotoxicity) against eukaryotic cells, so the cold-pressed oils can be considered safe.

## Figures and Tables

**Table 1:** Tested cold-pressed oils.

Origin		I C	II C	III C	IV A	V A	VI C	VII A	VIII C	IX A
Oil										
*Cannabis sativa* L. **(hemp oil)**		1	2		3	4	5	6	7	8
*Cannabis sativa* L. ECO **(hemp oil)**				9						
*Silybum marianum***(L.) Gaertner**. (milk thistle oil)		10	11		12	13	14	15	16	
***Silybum marianum* (L.) Gaertner ECO** (milk thistle oil)				17						
*Linum usitatissimum* L. (linseed oil)		18	19		20	21	22	23		
*Linum usitatissimum* L. ECO (linseed oil)				24						
*Linum flavum* L. (golden linseed oil)		25								
*Brassica napus* L. **(canola oil)**		26				27	28	29		
*Brassica napus* L. ECO **(canola oil)**				30						
*Helianthus annus* L. (sunflower oil)		31				32	33	34		
*Cucurbita pepo* L. (pumpkin oil)		35	36			37	38			
*Cucurbita pepo* L. ECO (pumpkin oil)				39						
*Camelina sativa* **(L.) Crantz** (camelina oil)		40	41		42					
*Camelina sativa* **(L.) Crantz** ECO (camelina oil)				43						
*Nigella sativa* L. (black seed oil)		44	45	46		47		48		
*Nigella sativa* L. ECO (black seed oil)				49						
*Oenothera biennis* L. (evening primrose oil)		50	51	73				52		
***Oenothera* *biennis* L. ECO**				53						
*Cocos nucifera* L. (coconut oil)		54	55							
*Sesamum indicum* L. (sesame oil)		56	57							
*Sesamum indicum* L. black (black sesame oil)		58								
*Carthamus tinctorius* L. (safflower oil)		59				60				
*Papaver somniferum* L. (poppy seed oil)		61								
*Sinapis alba* L. (mustard oil)		62	63							
*Borago officinalis* L. (borage oil)		64	65							
*Salvia hispanica* L. (chia seed oil)			66	74						
*Prunus armeniaca* L. seeds (apricot kernel oil)			67							
*Trigonella foenum-graecum* L. (fenugreek oil)				68						
*Hippophae rhamnoides* L. (sea buckthorn oil)				69						
*Daucus carota* L. ECO (carrot seed oil)				70						
*Urtica dioica* L. ECO (nettle seed oil)				71						
*Rosa canina* L. ECO (rose seed oil)				72						
***Glycine max*** **(L.) Merr. ECO (soybean oil)**				75						
***Brassica napus* L with *Levisticum officinale* W.D.J. Koch extract**				76						

I, II, III …—oil manufacturer number, C—commercial company, A—amateur company, 1,2,3 … 76—sample number, ECO—according to the manufacturer, oils pressed exclusively from seeds from organic farming.

**Table 2:** The antimicrobial activity of selected oils.

Oil No.	Oil Description	MIC [%] MBC/MFC [%]				
		*S. aureus* ATCC 29213	*S. epidermidis* ATCC 12228	*E. faecalis* ATCC 29212	*E. coli* ATCC 25922	*C. albicans* ATCC 10231
1	*Cannabis sativa* L.	>25	>25	>25	>25	>25
		>25	>25	>25	>25	>25
3	*Cannabis sativa* L.	>25	>25	>25	>25	>25
		>25	>25	>25	>25	>25
9	*Cannabis sativa* L. ECO	>25	>25	>25	>25	>25
		>25	>25	>25	>25	>25
12	*Silybum marianum*	>25	>25	>25	>25	>25
		>25	>25	>25	>25	>25
17	*Silybum marianum* ECO	>25	>25	>25	>25	>25
		>25	>25	>25	>25	>25
25	*Linum flavum* L.	>25	>25	>25	>25	>25
		>25	>25	>25	>25	>25
27	*Brassica napus* L.	>25	>25	>25	>25	>25
		>25	>25	>25	>25	>25
39	*Cucurbita pepo* L. ECO	>25	>25	>25	>25	>25
		>25	>25	>25	>25	>25
44	*Nigella sativa* L.	0.09	0.19	0.19	>25	6.25
		0.39	0.19	12.5	>25	25
46	*Nigella sativa* L.	0.78	0.19	0.39	>12.5	6.25
		0.78	0.78	0.78	>12.5	12.5
48	*Nigella sativa* L.	<0.09	0.02	1.56	>12.5	0.78
		<0.09	0.09	1.56	>12.5	3.12
49	*Nigella sativa* L. ECO	0.04	0.04	0.39	>25	6.25
		0.19	0.04	6.25	>25	25
50	*Oenothera* L.	>25	>25	>25	>25	>25
		>25	>25	>25	>25	>25
53	*Oenothera* L.	>25	>25	>25	>25	>25
		>25	>25	>25	>25	>25
68	*Trigonella foenum-graecum* L.	>25	>25	>25	>25	>25
		>25	>25	>25	>25	>25
69	*Hippophae rhamnoides* L.	>25	>25	>25	>25	>25

MIC—minimum inhibitory concentration measured by broth microdilution method; MBC/MFC—minimum bactericidal/fungicidal concentration determined based on microbial culture on solid media.

## Data Availability

The authors declare that data supporting the conclusions of this study are available in the article and in the information files. Raw data files in other formats and information on cold-pressed oil producers are available from the corresponding author upon reasonable request.

## Supplementary Materials

The following supporting information can be downloaded at: https://www.mdpi.com/article/10.3390/molecules30071425/s1, Table S1: Macronutrients content in cold-pressed oils (mg/kg); Table S2: Micronutrient content in cold-pressed oils (mg/kg); Table S3: Antioxidant activity of cold-pressed oils.
