# Supplementary material for: Macro- and Microelement Composition, Antioxidant Activity, and Biological Effect of Cold-Pressed Edible Oils from Commercial and Amateur Companies
Source: Molecules. 2025 Mar 23;30(7):1425. doi: 10.3390/molecules30071425 (PMC11990336; doi:10.3390/molecules30071425)
Supplement: Supplementary file 1 [file molecules-30-01425-s001.zip › molecules-3485350-supplementary.pdf]

Macro- and microelements composition, antioxidant activity and biological effect of cold pressed edible oils from commercial and amateur companies in Poland

Jolanta Marciniuk, Beata Sadowska, Marzena Więckowska-Szakiel, Mateusz Borkowski, Jacek Zebrowski, Bronisław Głód, Kacper Marciniuk, Paweł Marciniuk

Tab. S1. Macronutrients content in cold pressed oils (mg/kg)

Producer	Oil no.	Ca	Mg	K	Na	P	S	Macronutrients together
IC	1	50.57	125.72	255.19	270.78	220.70	15.59	938.55
IIC	2	46.58	15.13	157.41	172.02	73.46	26.93	491.53
IVA	3	96.67	58.97	172.50	155.10	188.10	64.93	736.27
VA	4	222.16	42.09	73.99	177.17	70.92	58.20	644.53
VIC	5	175.36	31.11	76.21	154.25	48.28	25.45	510.66
VIIA	6	45.55	10.96	52.80	113.47	28.68	17.17	268.63
VIIIC	7	47.81	13.31	62.04	157.64	62.55	57.14	400.49
IXA	8	90.97	23.62	49.43	153.65	48.06	43.27	409.00
IIIC	9	132.31	25.53	43.86	146.11	41.59	26.58	415.98
Cannabis mean		100.89	38.50	104.82	166.69	86.93	37.25	535.07
IC	10	25.04	8.13	49.89	133.49	26.27	22.54	265.36
IIC	11	294.89	53.54	75.04	286.99	19.75	75.47	805.68
IVA	12	421.22	115.09	308.70	255.88	356.40	103.95	1561.24
VA	13	218.66	67.74	89.74	203.21	222.32	62.87	864.54
VIC	14	25.36	11.84	59.75	129.76	25.89	38.45	291.05
VIIA	15	166.15	48.14	85.83	239.99	75.57	60.05	675.73
VIIIC	16	42.80	5.07	50.17	153.29	21.53	78.93	351.79
IIIC	17	64.99	18.89	45.31	127.54	70.00	16.87	343.60
Silybium mean		157.39	41.05	95.55	191.27	102.22	57.39	644.87
IC	18	45.05	11.91	40.91	125.66	46.44	45.02	314.99
IIC	19	211.39	39.13	31.06	182.79	30.55	92.77	587.69
IVA	20	46.33	42.51	129.29	121.90	122.68	94.01	556.72
VA	21	23.50	7.67	20.90	125.76	20.09	20.12	218.04

VIC	22	37.66	9.86	26.32	122.47	46.23	104.37	346.91
VIIA	23	205.72	40.93	23.43	158.07	28.02	115.98	572.15
IIIC	24	36.15	9.76	16.39	111.11	18.83	90.75	282.99
Linum mean		86.54	23.11	41.19	135.39	44.69	80.43	411.36
IC	25	40.52	9.99	15.54	103.39	56.02	32.42	257.88
IC	26	177.58	32.87	21.17	143.01	27.18	27.89	429.70
VA	27	56.99	13.47	18.37	207.75	38.03	51.94	386.55
VIC	28	114.36	35.81	26.87	116.01	181.02	26.46	500.53
VIIA	29	196.20	44.57	21.00	203.25	29.91	48.27	543.20
IIIC	30	32.23	9.89	13.69	122.78	17.45	12.78	208.82
Brassica mean		115.47	27.32	20.22	158.56	58.72	33.45	413.76
IC	31	136.74	27.65	6.38	179.54	15.71	63.82	429.84
VA	32	181.20	38.75	16.31	165.12	30.31	76.69	508.38
VIC	33	26.15	10.98	16.72	107.35	45.13	32.13	238.46
VIIA	34	6.60	3.00	5.44	104.75	47.17	0.58	167.54
Helianthus mean		87.67	20.09	11.21	139.19	34.58	43.30	336.05
IC	35	132.34	155.27	146.20	187.49	523.31	14.00	1158.61
IIC	36	24.34	22.47	24.53	116.54	52.51	79.44	319.83
VA	37	162.55	76.93	13.35	140.42	40.51	53.21	486.97
VIC	38	151.17	37.86	17.47	163.65	33.28	50.44	453.87
IIIC	39	50.77	31.68	29.77	125.73	64.50	53.43	355.88
Cucurbita mean		104.23	64.84	46.26	146.77	142.82	50.10	555.03
IC	40	110.07	23.54	4.08	149.27	17.34	38.95	343.25
IIC	41	24.76	10.84	7.48	123.71	71.90	21.46	260.15
IVA	42	73.41	33.28	155.23	116.60	143.83	85.47	607.82
IIIC	43	55.88	15.11	8.83	123.49	27.27	44.97	275.55
Camelina mean		66.03	20.69	43.90	128.267	65.08	47.71	371.69
IC	44	129.08	36.38	162.35	126.32	118.45	93.32	665.90
IIC	45	31.17	10.87	9.54	121.38	34.12	55.55	262.63

IIIC	46	37.29	11.25	1.09	141.12	43.22	52.59	286.56
VA	47	26.53	14.53	21.28	282.51	162.18	34.43	541.46
VIIA	48	96.37	25.74	79.48	141.66	73.45	89.94	506.64
IIIC	49	30.87	11.35	2.22	127.06	34.58	38.79	244.87
Nigella mean		58.55	18.35	45.99	156.67	77.67	60.77	418.01
IC	50	73.06	18.21	7.45	165.08	18.17	39.12	321.09
IIC	51	16.55	6.94	1.65	113.18	51.16	41.16	230.64
VIIA	52	199.81	46.69	7.09	213.48	19.46	44.80	531.33
IIIC	53	101.24	24.36	8.98	129.99	20.91	38.72	324.20
IIIC	73	36.74	9.29	23.16	105.64	53.19	29.23	257.25
Oenothera mean		85.48	21.10	9.67	145.47	32.58	38.61	332.90
IC	54	32.06	90.05	163.38	127.37	261.9	142.93	817.69
IIC	55	40.91	29.14	32.44	138.18	99.10	27.48	367.25
Cocos mean		36.48	59.59	97.91	132.77	180.50	85.20	592.47
IC	56	36.31	9.17	16.81	168.12	19.03	47.1	296.54
IIC	57	25.74	31.12	24.91	111.11	114.54	36.38	343.80
IC	58	45.50	13.10	8.40	114.17	65.52	25.78	272.47
Sezamum mean		35.85	17.80	16.71	131.13	66.36	36.42	304.27
IC	59	16.96	6.77	11.79	125.97	56.83	3.23	221.55
VA	60	80.86	23.53	13.00	137.54	22.41	65.63	342.97
Carthamus mean		48.91	15.15	12.39	131.75	39.62	34.43	282.26
IC	61	9.76	5.94	3.94	97.7	18.82	66.54	202.70
IC	62	24.48	7.65	10.78	115.68	72.07	35.40	266.06
IIC	63	477.04	82.03	270.74	125.08	190.23	97.60	1242.72
Sinapis mean		250.76	44.84	140.76	120.38	131.15	66.50	754.39
IC	64	144.47	35.86	1.58	139.35	62.95	51.76	435.97
IIC	65	52.67	14.03	6.52	112.700	45.86	29.73	261.51
Borago mean		98.57	24.94	4.05	126.02	54.40	40.74	348.74
IIC	66	26.90	8.54	9.00	123.82	41.77	74.83	284.86

IIIC	74	30.41	7.43	21.00	112.94	36.28	62.70	270.76
Salvia mean		28.65	7.98	15.00	118.38	39.02	68.76	277.81
IIC	67	13.13	12.15	5.46	126.65	86.11	61.05	304.55
IIIC	68	129.87	20.27	29.53	95.87	18.29	79.94	373.77
IIIC	69	34.16	7.42	22.07	92.91	12.94	39.47	208.97
IIIC	70	148.81	24.44	19.52	94.64	30.21	49.63	367.25
IIIC	71	32.91	6.97	21.67	113.69	30.19	16.90	222.33
IIIC	72	125.18	25.45	14.00	124.74	16.17	4.38	309.92
IIIC	75	5.96	5.20	23.87	98.65	16.63	59.57	209.88
IIIC	76	23.52	8.18	25.79	98.36	26.3	69.59	251.74

The names of the oils are given in Table 1 and Table S3

Tab. S2. Micronutrient content in cold pressed oils (mg/kg)

Producer	Oil no.	Fe	Zn	Mn	As	Cu	Cd	Pb	Mo	Cr	Se	Micro-nutrients together
IC	1	2.33	2.54	1.11	0.001	**0.420**	.	**0.213**	0.018	0.043	0.006	6.680
IIC	2	2.51	3.97	1.86	0.004	**0.634**	0.009	**0.509**	0.070	0.057	0.024	9.646
IVA	3	**7.15**	8.79	1.07	0.005	**0.763**	0.005	**0.931**	0.050	0.050	0.021	18.847
VA	4	**5.48**	3.65	2.18	0.004	**0.409**	.	**0.460**	0.049	0.081	0.010	12.326
VIC	5	4.04	3.13	0.82	0.002	**0.488**	.	**0.144**	0.036	0.052	0.007	8.722
VIIA	6	3.59	3.202	1.25	0.004	**0.591**	0.001	.	0.044	0.038	0.018	8.731
VIIIC	7	3.29	3.32	1.49	0.005	**0.926**	.	.	0.051	0.065	0.015	9.160
IXA	8	3.05	2.67	1.87	0.003	**0.698**	0.003	.	0.053	0.035	0.017	8.399
IIIC	9	2.65	7.99	1.37	0.005	**0.739**	.	.	0.057	0.047	0.019	12.89
Cannabis mean		3.79	4.37	1.45	0.003	0.630	0.002	0.251	0.048	0.052	0.015	10.599
IC	10	2.60	2.64	.	0.006	**0.491**	.	.	0.070	0.053	0.033	5.903
IIC	11	1.43	1.42	0.33	0.002	**0.833**	.	**0.191**	0.030	0.037	0.006	4.28
IVA	12	**8.69**	6.88	1.89	0.003	**1.079**	.	**0.796**	0.069	0.066	0.019	19.495
VA	13	**5.49**	4.93	1.72	0.005	**0.599**	.	**0.152**	0.064	0.061	0.030	13.050

VIC	14	2.38	4.72	0.10	0.005	0.398	.	.	0.065	0.032	0.027	7.727
VIIA	15	4.06	2.49	0.93	0.005	**0.425**	.	.	0.079	0.052	0.026	8.075
VIIIC	16	2.22	2.28	0.10	0.005	**0.789**	0.009	.	0.091	0.040	0.034	5.575
IIIC	17	4.96	5.31	0.88	0.003	0.309	.	.	0.041	0.048	0.018	11.562
Silybium mean		3.98	3.84	0.74	0.004	0.615		0.142	0.063	0.049	0.024	9.458
IC	18	3.16	3.08	0.02	0.005	**0.531**	.	.	0.071	0.039	0.023	6.932
IIC	19	**5.70**	3.21	.	0.005	**0.654**	.	.	0.069	0.037	0.014	9.686
IVA	20	2.26	3.37	0.70	0.004	0.382	.	.	0.049	0.041	0.021	6.820
VA	21	1.82	2.32	.	0.003	0.273	.	.	0.048	0.043	0.014	4.525
VIC	22	2.75	4.17	0.03	0.004	0.334	.	.	0.078	0.047	0.024	7.438
VIIA	23	2.30	2.50	.	0.002	0.226	.	.	0.060	0.052	0.013	5.158
IIIC	24	3.02	2.14	0.02	0.005	0.284	.	.	0.046	0.045	0.021	5.580
Linum mean		3.00	2.97	0.11	0.004	0.383			0.060	0.043	0.019	6.591
IC	25	3.18	4.21	0.06	0,003	0.221	.	**0.462**	0.043	0.047	0.014	8,27
IC	26	3.84	2.73	0.08	0.004	0.229	.	0.040	0.063	0.047	0.015	7.050
VA	27	2.22	2.48	0.07	0.003	0.243	.	0.044	0.047	0.047	0.015	5.177
VIC	28	4.59	3.09	0.59	0.002	**0.843**	.	**0.685**	0.019	0.053	0.004	9.874
VIIA	29	2.58	3.40	0.04	0.003	**0.785**	.	.	0.055	0.046	0.017	6.928
IIIC	30	2.53	2.30	0.02	0.003	**0.569**	.	.	0.079	0.038	0.021	5.567
Brassica mean		3.16	2.80	0.16	0.003	0.534		0.154	0.053	0.047	0.014	6.919
IC	31	2.39	2.51	.	0.003	0.175	.	.	0.031	0.051	0.007	5.163
VA	32	2.30	3.86	.	0.003	0.272	.	**0.136**	0.042	0.046	0.013	6.666
VIC	33	3.03	3.43	0.30	0.005	0.245	.	**0.221**	0.065	0.042	0.026	7.361
VIIA	34	2.23	2.61	.	0.003	0.185	.	**0.263**	0.031	0.038	0.013	5.369
Helianthus mean		2.49	3.10		0.003	0.219		0.155	0.042	0.044	0.015	6.065
IC	35	**6.13**	1.81	0.77	0.005	0.311	.	.	0.062	0.054	0.021	9.168
IIC	36	3.99	4.76	0.12	0.003	0.161	.	.	0.052	0.044	0.015	9.138
VA	37	3.12	1.39	0.02	0.003	0.185	.	**0.224**	0.061	0.067	0.016	5.082
VIC	38	2.69	2.60	0.07	0.004	0.149	.	.	0.038	0.041	0.013	5.610

IIIC	39	3.17	2.31	0.24	0.003	0.204	.	.	0.048	0.039	0.017	6.038
Cucurbita mean		3.82	2.58	0.24	0.003	0.202			0.052	0.049	0.016	6.962
IC	40	2.05	1.48	.	0.004	0.101	.	.	0.042	0.050	0.008	3.734
IIC	41	2.98	3.90	0.06	0.005	0.150	.	.	0.049	0.047	0.015	7.201
IVA	42	2.37	3.30	1.08	0.004	0.330	.	.	0.057	0.058	0.023	7.225
IIIC	43	**7.26**	4.41	0.09	0.004	0.167	.	.	0.072	0.045	0.023	12.079
Camelina mean		3.667	3.67	0,31	0.004	0.188			0.055	0.050	0.018	7.560
IC	44	4.39	3.95	0.20	0.004	0.179	.	.	0.056	0.047	0.017	8.843
IIC	45	**5.24**	3.30	0.06	0.004	0.249	.	.	0.048	0.049	0.027	8.978
IIIC	46	3.80	5.78	0.09	0.003	0.230	.	.	0.043	0.053	0.018	10.018
VA	47	1.99	1.38	0.15	0.004	0.119	.	.	0.061	0.051	0.022	3.775
VIIA	48	**5.50**	2.81	0.59	0.005	0.616	.	.	0.068	0.039	0.027	9.652
IIIC	49	2.35	2.02	0.02	0.004	0.133	.	.	0.072	0.050	0.026	4.672
Nigella mean		3.877	3.88	0.19	0.004	0.254			0.058	0.048	0.023	7.656
IC	50	2.09	2.47	0.14	0.002	**0.817**	.	.	0.038	0.050	0.011	5.622
IIC	51	4.89	4.03	0.28	0.004	0.306	.	.	0.062	0.050	0.021	9.638
VIIA	52	2.50	1.65	0.04	0.004	1.125	.	.	0.066	0.051	0.015	5.453
IIIC	53	3.31	2.89	0.30	0.003	0.146	.	.	0.045	0.061	0.011	6.769
IIIC	73	3.42	2.35	0.38	0.002	0.170	.	.	0.052	0.042	0.010	6.417
Oenothera mean		3.241	3.24	0.23	0.003	0.513			0.0537	0.0517	0.0137	6.770
IC	54	1.13	3.65	3.03	0.003	0.239	.	.	0.071	0.056	0.004	8.179
IIC	55	3.99	1.65	0.44	0.005	**0.767**	.	.	0.045	0.050	0.015	6.961
Cocos mean		2.559	2.56	1.73	0.004	0.503			0.058	0.053	0.010	7.570
IC	56	2.77	2.37	.	0.005	0.450	.	.	0.063	0.050	0.015	5.721
IIC	57	3.68	4.30	0.27	0.003	**0.666**	.	.	0.032	0.041	0.025	9.019
IC	58	3.16	3.12	0.07	0.003	0.251	.	.	0.030	0.037	0.009	6.674
Sezamum mean		3.202	3.20	0.11	0.004	0.456			0.042	0.043	0.017	7.138
IC	59	3.28	4.73	.	0.003	**0.558**	.	.	0.035	0.055	0.013	8.677
VA	60	2.71	0.69	0.04	0.005	**1.143**	.	.	0.089	0.046	0.032	4.752

Carthamus mean		2.994	2.99	-	0,004	0.851			0.062	0.050	0.022	6.694
IC	62	2.74	2.27	0.28	0.004	**0.814**	.	.	0.052	0.045	0.018	6.226
IIC	63	2.67	4.55	7.12	0.006	0.291	.	.	0.071	0.016	0.028	14.748
Sinapis mean		2.704	2.70	3.70	0.005	0.552			0.061	0.030	0.023	10.487
IC	64	2.75	1.21	0.30	0.005	**0.887**	.	.	0.066	0.047	0.021	5.286
IIC	65	2.60	6.29	0.24	0.003	**0.998**	.	**0.246**	0.055	0.042	0.015	10.480
Borago mean		2.674	2.67	0.26	0.004	0.943			0.061	0.045	0.018	7.760
IIC	66	2.73	3.46	.	0.005	0.126	.	.	0.076	0.049	0.028	6.467
IIIC	74	2.38	2.08	0.14	0.004	0.103	.	.	0.064	0.041	0.020	4.833
Salvia mean		2.553	2.55	-	0.004	0.115			0.070	0.045	0.024	5.579
IC	61	2.37	2.19	0.11	0.007	**0.825**	.	.	0.074	0.052	0.035	5.669
IIC	67	2.75	4.48	0.14	0.004	**1.047**	.	**0.246**	0.058	0.052	0.024	8.804
IIIC	68	4.05	3.71	0.14	0.005	**1.283**	.	.	0.048	0.027	0.021	9.291
IIIC	69	4.14	3.13	0.14	0.005	0.235	.	.	0.082	0.042	0.022	7.801
IIIC	70	3.41	7.61	0.14	0.003	**0.545**	.	**0.550**	0.042	0.042	0.013	12.355
IIIC	71	3.03	3.11	0.14	0.003	**0.485**	.	**0.333**	0.039	0.045	0.013	7.208
IIIC	72	5.06	7.16	0.14	0.003	**0.708**	.	.	0.042	0.053	0.014	13.184
IIIC	75	1.84	3.88	.	0.005	0.079	.	.	0.059	0.039	0.031	5.925
IIIC	76	2.07	2.81	.	0.006	0.169	.	.	0.077	0.049	0.025	5.200

Concentrations higher than the permissible limits are marked in bold. The names of the oils are given in Table 1 and Table S3

Tab. S3. Antioxidant activity of cold-pressed oils

Oil name	oil number	antioxidant activity in %
Cannabis sativa	1	55.29
	2	56.86
	3	61.68
	4	61.50
	5	66.35
	6	72.71
	7	60.65
	8	45.21

	9	54.71
	mean	59.44
Silybum marianum	10	42.78
	11	42.95
	12	42.98
	13	46.70
	14	41.21
	15	43.73
	16	38.04
	17	39.39
	mean	42.22
Linum usitatissimum	18	49.51
	19	53.06
	20	50.05
	21	52.04
	22	48.63
	23	50.65
	24	52.27
	mean	50.89
Linum flavum	25	45. 63
Brassica napus	26	58.90
	27	58.53
	28	58.39
	29	54.72
	30	56.70
	mean	57.45
Helianthus annuus	31	58.98
	32	48.18
	33	44.03
	34	62.43
	mean	53.41
Cucurbita pepo	35	41.67
	36	57.72
	37	58.41
	38	49.65
	39	56.41
	mean	52.77
Camelina sativa	40	56.96
	41	55.72
	42	53.11
	43	57.22

	mean	55.75
Nigella sativa	44	93.91
	45	95.83
	46	95.77
	47	94.55
	48	95.63
	49	96.04
	mean	95.29
Oenothera biennis	50	47.87
	51	60.06
	52	59.90
	53	63.57
	73	66.86
	mean	59.65
Cocos nucifera	54	4.14
	55	6.24
	mean	5.19
Sezamum indicum	56	34.92
	57	41.86
	58	42.03
	mean	39.60
Carthamus tinctorius	59	45.50
	60	49.58
	mean	47.54
Sinapis alba	62	46.58
	63	49.43
	mean	48.01
Borago officinalis	64	55.63
	65	67.55
	mean	61.59
Salvia hispanica	66	45.49
	74	40.87
	mean	43.18
Papaver somniferum	61	31.51
Prunus armeniaca	67	50.61
Trigonella foenum-graecum	68	56.44
Hippophae rhamnoides	69	44.99
Daucus carota	70	56.81
Urtica dioica	71	53.56
Rosa canina	72	74.22
Glycine max	75	75.10

Brassica napus with *Levisticum officinale*	76	46.19
